# Transcriptomic Analyses Reveal B-Cell Translocation Gene 2 as a Potential Therapeutic Target in Ovarian Cancer

**DOI:** 10.3389/fonc.2021.681250

**Published:** 2021-08-17

**Authors:** Jia Wang, Haonan Li, Liang Wang, Jing Zhang, Man Li, Liang Qiao, Jun Zhang, Likun Liu, Cuili Zhang, Jingchun Gao, Weiling Li

**Affiliations:** ^1^Department of Biotechnology, College of Basic Medical Sciences, Dalian Medical University, Dalian, China; ^2^Laboratory Animal Center, Dalian Medical University, Dalian, China; ^3^Department of Hematological Malignancies Translational Science and the Gehr Family Center for Leukemia Research, Beckman Research Institute, City of Hope Medical Center, Duarte, CA, United States; ^4^Department of Pathology, Dalian Medical University, Dalian, China; ^5^Department of Obstetrics and Gynecology, First Affiliated Hospital of Dalian Medical University, Dalian, China; ^6^Liaoning Key Laboratory of Hematopoietic Stem Cell Transplantation and Translational Medicine, Dalian, China

**Keywords:** BTG2, cell cycle, migration, ovarian cancer, proliferation

## Abstract

Ovarian cancer is the most common and aggressive type of tumor of the female reproductive system. Two factors account for this detrimental clinical presentation: (i) the lack of early detection methods and (ii) the inherently aggressive nature of this malignancy. Currently, transcriptomic analyses have become important tools to identify new targets in different cancer types. In this study, by measuring expression levels in ovarian cancer samples and stem cell samples, we identified 24 tumor suppressor genes consistently associated with poor prognosis. Combined results further revealed a potential therapeutic candidate, BTG2, which belongs to the antiproliferative gene family. Our results showed that BTG2 expression regulated ovarian cancer cell proliferation *via* G1/S phase cell cycle arrest by regulating Cyclin D1, CDK4, p-AKT, and p-ERK expression. BTG2 also inhibited cell migration by modulating MMP-2 and MMP-9 expression. Furthermore, xenograft models confirmed a growth inhibitory effect of BTG2 in ovarian cancer *in vivo*. BTG2 was significantly associated with ovarian cancer FIGO stage and grade in the clinic. Our findings indicated that BTG2 exerts a suppressive impact on ovarian cancer and could be a potential biomarker.

## Introduction

Ovarian cancer is the leading and most common female reproductive malignancy among female patients, with 25,000 deaths per year in China alone (1). Most diagnoses occur in the late stage, so patients have missed the optimal window for surgery. The five-year survival rate of these patients is only approximately 25% (2). Compared with patients diagnosed with late-stage disease, patients diagnosed with early-stage disease have a considerably more favorable prognosis, although different prognoses still exist among patients with similar clinical characteristics (3, 4). Therefore, an improved understanding of the genetic and molecular heterogeneity among patients and identification of potential biomarkers to diagnose and monitor treatment for ovarian cancer at an earlier stage are urgently needed.

With the development of sequencing technology, genomic and transcriptomic analyses have become important tools to identify new therapeutic strategies. The Cancer Genome Atlas (TCGA) has been instrumental in improving the classification and identification of tumor drivers in many studies (5–7). For example, differentially expressed genes (DEGs) regulated at the gene transcription level are implicated in diverse biological processes based on TCGA (8–10). Due to the importance of DEGs in cancer research, the roles of DEGs as biomarkers and drivers of tumor oncogenesis and suppression have been identified in ovarian cancer (11, 12). Because novel therapeutic strategies based on these findings have not been developed, it is necessary to investigate additional pathways of gene deregulation in ovarian cancer. Equally important is the study of ovarian cancer stem cells, which play an important role in the development, invasion, metastasis, and drug resistance recurrence of ovarian cancer (13). A growing number of inhibitors against stem cell characteristics have been identified (14). Traditional tumor cytoreductive surgery combined with cisplatin systemic chemotherapy has a certain effect on reducing tumor volume and alleviating clinical symptoms, but residual epithelial ovarian cancer stem cells after treatment can rebuild tumor tissue in a short time, which is the root cause of ovarian cancer recurrence and refractory (15).Therefore, identifying regulators that maintain ovarian cancer stem cell phenotypes and/or contribute to their survival is critical for designing novel therapeutic strategies (16, 17).

To identify new targets for therapy, we conducted a combination of transcriptomic analyses based on TCGA database and ovarian cancer stem cell gene expression files and then performed biological validation *in vitro* and *in vivo*. B cell translocation gene 2 (BTG2) belongs to the antiproliferative gene family. Our data revealed that lower expression of BTG2 in ovarian cancer patients was associated with tumor progression and shortened survival. BTG2 inhibited ovarian cancer cell proliferation and migration, blocked the ovarian cancer cell cycle and enhanced the cisplatin sensitivity of ovarian cancer cells. Furthermore, knockdown of BTG2 expression contributed to ovarian tumor formation in a mouse xenograft assay. These results suggested that BTG2 is a tumor suppressor and could be used as a potential biomarker for ovarian cancer.

## Materials and Methods

### Gene Expression Analysis of Ovarian Cancer RNA-Seq Data

Read counts per gene of 419 samples of ovarian cancer from TCGA and 88 samples of normal ovaries from GTEx were downloaded from the UCSC Xena portal (**Table S1**) (18). Differential expression analysis was performed using DESeq2 comparing the tumor samples to the normal samples (19). All downregulated genes that were differentially expressed between the tumor and normal samples as shown by a Benjamini-Hochberg corrected p-value <0.05 and log2-fold change <1 were selected.

### Gene Expression Analysis of Ovarian Cancer Stem Cell Microarray Data

Microarray data between the side population (SP) and main population (MP) isolated from fresh ascites obtained from women with high-grade advanced stage papillary serous ovarian adenocarcinoma were obtained from Vinod (**Table S2**) (20). Data were normalized using Robust Multichip Average. DEGs between the SP and MP samples were identified using the LIMMA package (21) with p-value <0.05 and log2-fold change ≥|1|.

### Survival Analysis and Grade Analysis

Kaplan-Meier plotter was used to evaluate whether increased expression of the selected genes was associated with a poorer prognosis in ovarian cancer (22). Samples with increased expression of selected genes were compared to all other samples. Kaplan-Meier survival curves with overall survival were established and then compared using a log-rank test.

Normalized expression data from grade I, II and III-IV ovarian cancer (10 grade I, 101 grade II, 607 grade III-IV) were downloaded from the Xena portal (18). Expression levels of 24 selected genes in samples with different grades were compared using one-way ANOVA with Tukey’s multiple comparisons test.

### Mutation and CNA Analysis

All 311 ovarian cancer samples with exome sequencing and copy-number alterations (CNA) data available in cBioPortal (23) were evaluated. The gene set containing 24 selected tumor suppressors was analyzed. All samples containing at least one alteration in one or more of these selected were identified and presented. A simulation with 100,000 random sets of 24 out of 94 tumor suppressors and the hypergeometric test were performed to determine if our selected gene set presented enrichment for mutation and CNA. Mutation and CNA data for all tumor suppressors were retrieved from cBioPortal using the CDGS-R package (24).

### Functional Annotation

Functional annotation analyses (Gene Ontology and KEGG pathways) were performed using DAVID (25) using *Homo sapiens* genes as background. Terms with Benjamini-Hochberg corrected p-values<0.05 were determined to be enriched. The STRING database was used to analyze the protein-protein interaction (PPI) networks of the top potential tumor suppressors (26). The results were visualized using Cytoscape version 3.7.1, a software platform used to visualize complex networks (27).

### Patients, Tissue Specimens, and Histopathological Data

A total of 38 ovarian samples, including 30 patients with radically resected stage I to IV ovarian cancer and 8 normal ovarian tissues, were collected under the instructions of The Code of Ethics of the World Medical Association during the period of 2016 to 2017 from the Pathology Department, The First Affiliated Hospital of Dalian Medical University. All patients formally and voluntarily gave their consent for the study. The diagnosis of BTG2 confirmed in the tissue samples and the histologic subtypes and stages of tumors were identified using the World Health Organization classification. This study protocol was approved by The Ethical Research Committee of the First Affiliated Hospital of Dalian Medical University.

### Cell Culture

The human ovarian cancer cell lines SKOV3 (RRID: CVCL_0532) and A2780 (RRID : CVCL_0134) (American Type Culture Collection, Rockville, MD, USA) were maintained in DMEM (Gibco BRL, Grand Island, NY, USA), supplemented with 10% fetal bovine serum (TransGen Company, Beijing, China) and cultured at 37°C in 5% CO2. All experiments were performed with mycoplasma-free cells.

### Cell Transfection

The vector containing the BTG2 DNA sequence and shRNA targeting BTG2 were constructed by GenePharma Company (Shanghai, China). The ovarian cancer A2780 and SKOV3 cell lines were transfected with plasmids using Lipofectamine™ 2000 (Invitrogen, Carlsbad, CA, USA) according to the manufacturer’s recommended protocol, and stable cell lines were subsequently established by G418 (Sigma-Aldrich). Ovarian cancer cells stably overexpressing BTG2 are referred to as the GV-BTG2 group, cells stably transfected with the negative control are referred to as the GV-COL group, and cells stably transfected with shRNA targeting BTG2 or the negative control are considered the shBTG and shCOL groups. BTG2 expression was assessed by Western blotting analysis.

### Quantitative Real Time PCR

Total RNA was isolated from tissue samples or cultured cells using the FFPE total RNA purification kit (GenePharma, Suzhou, China) according to the manufacturer’s instructions. Total RNA (1 μg) was reverse-transcribed into first-strand complementary DNA (cDNA) with the FastQuant RT Kit (Tiangen, Beijing, China). qPCR was performed with a SuperReal PreMix Plus SYBR Green PCR kit (Tiangen, Beijing, China) using a StepOne Real-Time PCR machine (Thermo Fisher Scientific, Rockford, IL, USA). Experiments were performed in triplicate. The average threshold cycle (Ct) value of BTG2 was normalized to the average Ct value of β-actin. The specific primers used in the qPCR were as follows: for BTG2, 5′-CGGAATTCCGCGACATGAGCCACGGGAAG-3′ and 5′- GCAGCTCGAGGCCTAGCTGGAGACTGCCAT-3′; and for β-actin, 5′- AGAAAATCTGGCACCACACC-3′ and 5′- TAGCACAGCCTGGATAGCAA-3′.

### Western Blotting

Cell lysates were prepared in RIPA buffer (Beyotime, China) and quantified by using a BCA assay kit (Thermo Fisher Scientific). Equivalent amounts of each sample were resolved by SDS-PAGE and analyzed by Western blotting using the same method as previously described (28). Antibodies against BTG2, ERK, AKT, p-AKT, CDK2, CDK4, MMP2, MMP9 and β-actin were obtained from Proteintech Group, Inc. (Chicago, USA). The antibody against cyclin D1 was purchased from Cell Signaling Technology (MA, USA), and the antibodies against cyclin E1 and p-ERK were purchased from Bioss (Boston, USA).

### Cell Proliferation Assays

Cells were seeded in 96-well plates at 1×10^4^ cells/well and incubated for 1, 2 or 3 days. Then, 20 μl of MTT (5 mg/mL in PBS) was added to each well and incubated for 4 h. The MTT solution was removed, and the formazan was dissolved in 150 μl of DMSO. Absorbance of the solution was measured using a Multiskan Ascent plate reader at 540 nm wavelength.

### Colony Formation Assay

Three hundred cells per well in 6-well plates were seeded before treatment. Then, 2 ml of medium with or without cisplatin (2 μM) was added to each well. Cells were fixed after 2 weeks using methanol and stained using crystal violet (Sigma, St. Louis, MO, USA) dissolved in 10% ethanol. The number of stable colonies was obtained by counting. Colonies were defined as a minimum of 50 cells in a cell group.

### Cell Cycle Analysis

Cell cycle distribution was examined by propidium iodide (PI) staining and flow cytometry. Briefly, cells were collected, washed with PBS and fixed with ice-cold 70% ethanol overnight at 4°C. The fixed cells were then washed with PBS and incubated with staining buffer containing PI and RNase for 1 h at 37°C. The cell cycle was examined using a FACSCalibur flow cytometer (BD Biosciences, San Jose, CA, USA). The percentage of cells in each phase was determined by ModFitLT V4.1.

### Transwell Migration Assays

Cell migration was quantified by the Transwell assay. A total of 2×10^4^ cells in 200 μl of serum-free DMEM were added to each upper chamber, and DMEM with 10% FBS was added to the lower chamber as a chemoattractant. After 24 h incubation at 37°C, the underside of the membrane was stained with 0.1% crystal violet for 10 mins and counted on average for six random fields under a microscope at 100× magnification field.

### Immunohistochemistry Analysis and Scoring System

Immunohistochemical staining was conducted to detect BTG2 protein expression using SPlink detection kits (ZSGB-BIO, Beijing, China) in paraffin embedded specimens of ovarian cancer tissues. The slides were dewaxed with xylene and gradually hydrated. Subsequently incubated with endogenous peroxidase blocker at room temperature for 40 min and washed thrice with PBS. All tissue slides were blocked with goat serum at room temperature for 30 min and incubated with the Anti-BTG2 antibody (diluted 1:200 with PBS) overnight at room temperature. After incubation with secondary biotinylated antibody Goat Anti-Mouse IgG at room temperature for 13 min, the sections were then rinsed with PBS. Developed with chromogen for 100 sec at room temperature and rinse in running tap water for 100 sec. Final slides were rinsed with distilled water, counterstained with hematoxylin, dehydrated, and mounted.

The photo of immunohistochemical staining was taken under the microscope and results were interpreted independently by two pathologists. Five fields were selected randomly under a high-power microscope (magnification, x200; Olympus BX41, Tokyo, Japan), followed by scoring based on the proportion and intensity of positive cells. The: proportion of positive cells was scored as 0 (<25%), 1 (26-50%), 2 (51-75%) or 3 (>75%). The staining intensity was scored as 0 (negative), 1 (weak), 2 (moderate) 3 (strong) or 4 (strongest). The final score of each slide was assessed by multiplying the scores for proportion and intensity. The final score≥6 points indicated positive expression.

### Xenograft Models

Female athymic nude mice (BALB/c-nu/nu) (Institute of Genome Engineered Animal Models for Human Disease, Dalian Medical University), approximately 6-8 weeks old and weighing 15-18 g were breeding in specific-pathogen-free(SPF) conditions. All animal experiments were approved by Experimental Animal Ethics Committee of Dalian Medical University (No. AEE19104), Mice xenograft models were established by subcutaneous injection of 4×10^6^ scrambled shRNA transduced A2780 cells and 4×10^6^ shBTG2 transduced A2780 cells into the right posterior ventral side of ten nude mice. Tumor growth was calculated with the length (L) and width (W) of tumors by vernier calipers every 2 days after injection, and the tumor volume (V) was calculated by the formula V = (L×W×W)/2. All the mice were sacrificed 21 days after injection. The xenograft tumors were recovered and weighed. BTG2 expression in the xenograft tumors was analyzed by immunohistochemistry staining.

### Statistical analysis

All experiments were independently repeated three times. The results are presented as the means ± SD. All statistical analyses were executed using R. All the differences results in a p-value < 0.05 were considered significant.

## Results

### Identification of Aberrantly Expressed Tumor Suppressors in Ovarian Cancer

**Figure 1:**
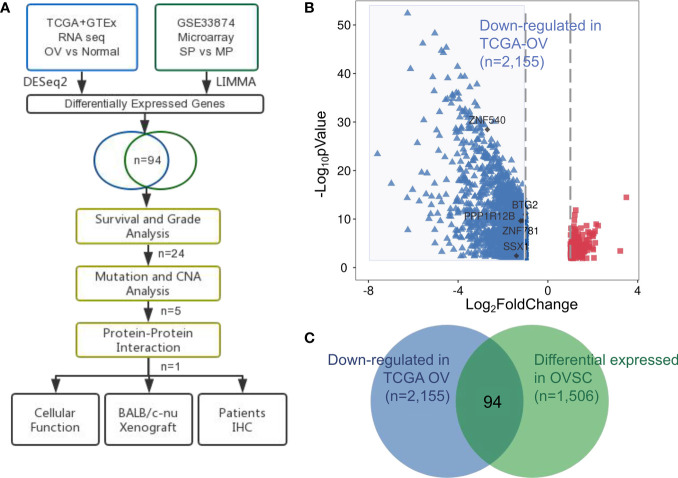
Identification of aberrantly expressed tumor suppressors in ovarian cancer. **(A)** Flow chart of methodologies applied in the current study. **(B)** Volcano plot of fold changes in the expression of the DEGs in ovarian cancer patients. Red rectangle dots, significantly upregulated genes, blue triangle dots, significantly downregulated genes and black dots, hub genes. **(C)** Venn diagram of the 94 significantly co-expressed genes compared with down-regulated in ovarian cancer and differential expressed in OVSC.

To identify potential targets involved in ovarian cancer, we examined both ovarian cancer RNA sequencing (RNA-Seq) samples and ovarian cancer stem cell gene expression profiles, as shown in **Figure 1A**. We obtained RNA-Seq data for 419 ovarian cancer samples and compared them with 88 normal ovary samples from the UCSC Xena database (see *‘Materials and Methods’*; **Tables S1** and **S2**). The identification of 214 upregulated and 2,155 downregulated genes in the tumor samples compared to the normal samples is shown in **Figure 1B** (detailed in **Table S3**).

We examined the microarray dataset of Vinod et al. (20) to identify differentially expressed genes between the SP and MP isolated from fresh ascites obtained from women with high-grade advanced stage papillary serous ovarian adenocarcinoma. This analysis revealed a total of 1,048 upregulated and 458 downregulated genes in ovarian cancer stem cells (**Table S4**).

We next focused on the identification of tumor suppressors that were closely related to tumor progression and tended to be attractive targets in therapeutic contexts. The results from both transcriptomic studies were merged: 94 tumor suppressor genes were identified in both ovarian cancer and ovarian cancer stem cell samples (**Table S5**), in **Figure 1C** representing a significant overlap (p-value= 0.0004; hypergeometric test).

### Development of 94 Tumor Suppressors in Survival and Grade Analysis

**Figure 2:**
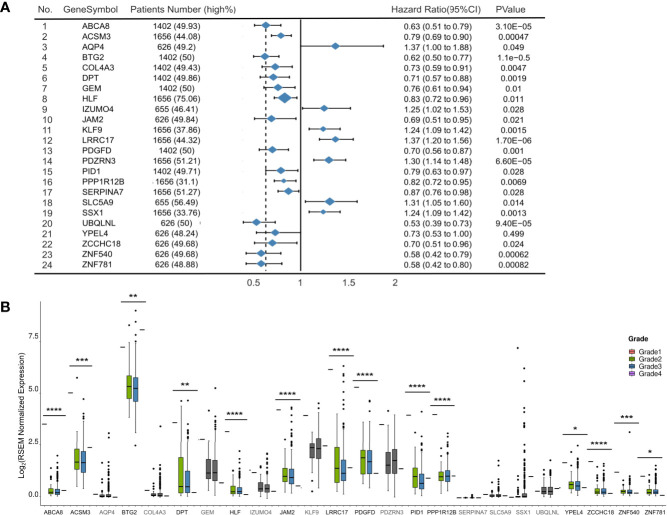
Development of 94 tumor suppressors in survival and grade analysis. **(A)** Forest plot of 24 significantly expression genes. Hazard ratio and p-value are calculated by using a Kaplan-Meier analysis. **(B)** Boxplots (with median) showing the 14 out of 24 genes with expression levels in samples from TCGA significantly related to tumor grade. *p < 0.05, **p < 0.01, ***p < 0.001 and ****p < 0.0001.

We established Kaplan-Meier survival curves to determine whether 94 tumor suppressors exhibit an association with poor prognosis in ovarian cancer (detailed in **Table S6**). Twenty-four of the 94 tumor suppressors showed an association with overall survival reduction when overexpressed (p-value < 0.05; log-rank test). **Figure 2A** presents a summary of the 24 selected tumor suppressors and their results in survival analysis.

To explore the roles of these 24 selected tumor suppressors in an additional context, we evaluated their expression levels in samples with different grade from TCGA (**Figure 2B**). In general, 14 of 24 tumor suppressors exhibited lower expression levels in higher grade ovarian cancer samples than in lower grade samples (p-value<0.05; Wilcoxon rank-sum test and **Table S7**).

### BTG2 as a Potential Tumor Suppressor Candidate in Ovarian Cancer

**Figure 3:**
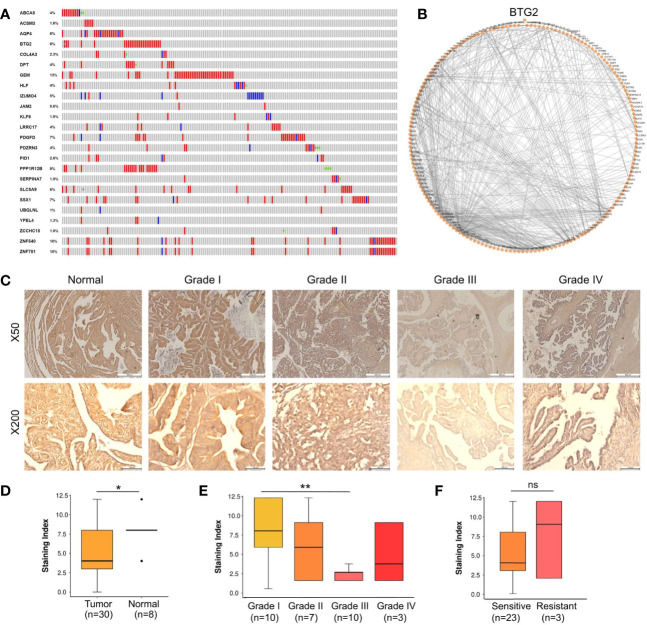
BTG2 as a potential tumor suppressor candidate in ovarian cancer. **(A)** Gene mutations and copy-number alterations analysis of 24 genes were analyzed using cBioPortal. Tumor samples are shown in columns. **(B)** Diagram of the individual modules and their interactions of BTG2 module. Circles (nodes) represent the 128 modules, with the circle size proportional to module size. Edges connect modules that share PPIs. **(C)** Normal samples showed normal fallopian tube epithelium tissues and Stage I-IV samples showed ovarian cancer tissues. **(D)** BTG2 staining index was lower in ovarian cancer tissues compared to normal tissues in ovarian cancer patients. **(E)** BTG2 staining index of ovarian cancer tissues was relative to tumor grade. **(F)** Correlation between sensitive and resistant expression of ovarian cancer was analyze. *p < 0.05, **p < 0.01, ns is p-value.

We also evaluated whether the 24 tumor suppressors presented mutations and/or CNA in ovarian cancer samples from TCGA as shown in **Figure 3A**. Only 10% of the samples displayed alterations in at least one of our selected tumor suppressors. Mutation and CAN presented in 24 selected tumor suppressors displayed was not different from randomly selected RBP sets (p-value > 0.1; simulation with 100,000 sets of 24 randomly selected tumor suppressors).

The top 5 most mutated nodes (BTG2, PPP1R12B, SSX1, ZNF540 and ZNF781) were identified by PPI networks in the String database. Physiologically, proteins rarely function alone and instead function in networks. Cytoscape software was used to visualize the data, and the top 5 most mutated nodes were identified and evaluated by degree (BTG2: node degree=128; PPP1R12B: node degree=61; SSX1: node degree=44; ZNF540: node degree =17; ZNF781: node degree=18). PPI networks in ovarian cancer were constructed to identify the BTG2 gene (**Figure 3B**). Overall, BTG2, a member of the BTG/TOB (B-cell translocation gene/transducers of ErbB2) gene family, showed the highest node degree and exhibited the most consistent results in bioinformatics analysis.

A tissue cohort composed of tumor and adjacent normal tissues from 38 ovarian cancer patients was employed to address the expression alteration and clinical implications of BTG2. As shown in **Figures 3C, D**, BTG2 protein expression in ovarian tumor tissue samples (n=30) was significantly lower than that in normal ovarian tissue samples (n=8) (p<0.05). Furthermore, we found that BTG2 expression was related to tumor FIGO stage (p<0.001) and grade (p<0.01), as shown in **Table 1** and **Figure 3E**. Moreover, although we found that chemosensitive patients had lower expression of BTG2 than chemoresistant patients, there was no significant difference between the two groups (**Figure 3F** and **Table S8**).

**Table 1:** Clinical characteristics.

Variable	Total	Expression of BTG2		*P value*
		High(%)	Low (%)	
				0.0262*
Normal Ovary	8	7 (88)	1 (12)	
Ovarian Cancer	30	13 (43)	17 (57)	
Grade				0.0032**
I	10	8 (62)	2 (12)	
II	7	4 (31)	3 (18)	
III	10	0 (0)	10 (59)	
IV	3	1 (7)	2 (11)	
FIGO stage				0.0006***
I-II	17	12 (92)	5 (29)	
III-IV	13	1 (8)	12 (71)	
Histologic subtype				0.1542
Serous adenocarcinoma	22	7 (58)	15 (83)	
Mucinous adenocarcinoma	3	3 (26)	0 (0)	
Endometrioid adenocarcinoma	3	1 (8)	2 (11)	
Clear cell carcinoma	2	1 (8)	1 (6)	
CA125 at diagnosis				0.4693
<500U/ml	21	10 (77)	11 (65)	
≥500U/ml	9	3 (23)	6 (35)	
Age (years)				0.6379
<55	16	6 (50)	10 (59)	
≥55	13	6 (50)	7 (41)	
Chemotherapy sensitivity				0.3639
Sensitive	23	9 (82)	14 (93)	
Resistant	3	2 (18)	1 (7)	

Data was analyzed by Chi-square test.

Results were considered statistically significant at *p < 0.05, **p < 0.01,***p < 0.001.

FIGO international federation of gynecology and obstetrics.

### BTG2 Impacts Cell Proliferation, G1-Phase Arrest, and Cell Migration in Ovarian Cancer

**Figure 4:**
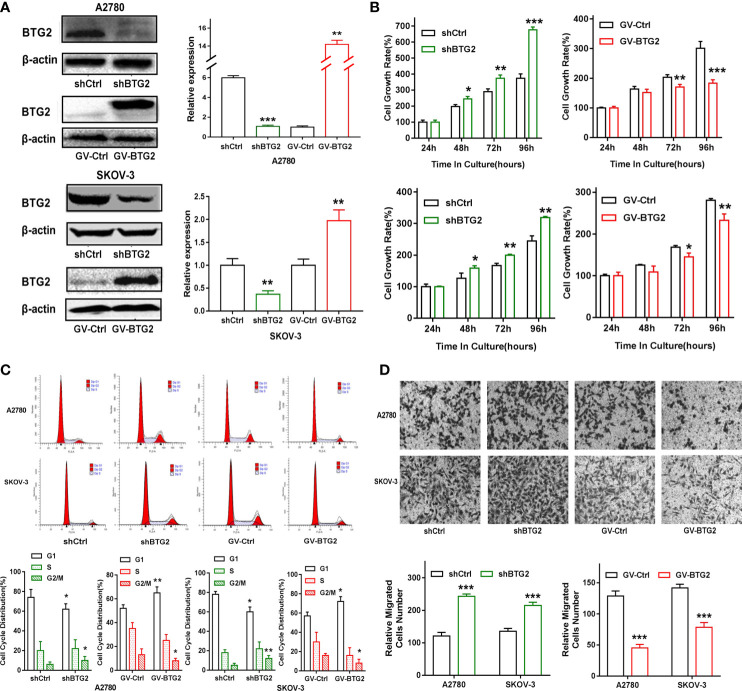
BTG2 inhibited ovarian cancer cell proliferation, blocked cell cycle progression and impacts cell migration. **(A)** Knockdown and Over-expression efficiencies were determined by Western blot in A2780 and SKOV3 cells. **(B)** Knockdown and Over-expression of BTG2 significantly reduced the proliferative capacities of A2780 and SKOV3 cells, as determined by MTT assay. **(C)** Cell cycle distribution was analyzed using flow cytometry and the percentage of G1 phase was significantly decreased in shBTG2 group compared to negative control group and increased in GV-BTG2 group compared to negative control group in both A2780 and SKOV3 cells. **(D)** The images represented cell migration into the underside of transwell membrane under microscope at 100×magnifcation field. A2780 and SKOV3 cells that crossed the transwell chamber of each group was counted and averaged. The experiments were repeated three times with three replicates each. *p < 0.05, **p < 0.01, ***p < 0.001.

To investigate the role of BTG2 in ovarian cancer, A2780 and SKOV-3 ovarian cancer cells were transfected with shBTG2 and GV-BTG2 plasmids (**Figure 4A**). MTT assays revealed that the growth rate of A2780 and SKOV-3 cells transfected with shBTG2 was significantly increased compared with that of the negative control, while the cells transfected with GV-BTG2 showed the opposite effect (**Figure 4B**). We further explored whether dysregulation of BTG2 inhibited ovarian cancer growth by modulating the cell cycle. The results showed that the percentage of G1 phase cells decreased in the shBTG2 ovarian cancer cells, while the percentage of S and G2 phase cells increased. However, the percentage of G1 phase cells increased in the GV-BTG2 ovarian cancer cells, while the percentage of S and G2 phase cells decreased (**Figure 4C**). The results above indicated that BTG2 arrested cells in G1 phase, which may contribute to inhibiting ovarian cancer growth.

In addition, we found that BTG2 knockdown significantly increased the *in vitro* migration of A2780 and SKOV-3 ovarian cancer cells, and BTG2 overexpression inhibited the migration of ovarian cancer cells, as shown in **Figure 4D**. These results confirmed that BTG2 acted as a tumor suppressor in ovarian cancer.

### BTG2 Prevents Cell Proliferation and Dysregulates the Cell Cycle Through the AKT and ERK-MAPK Signaling Pathways

**Figure 5:**
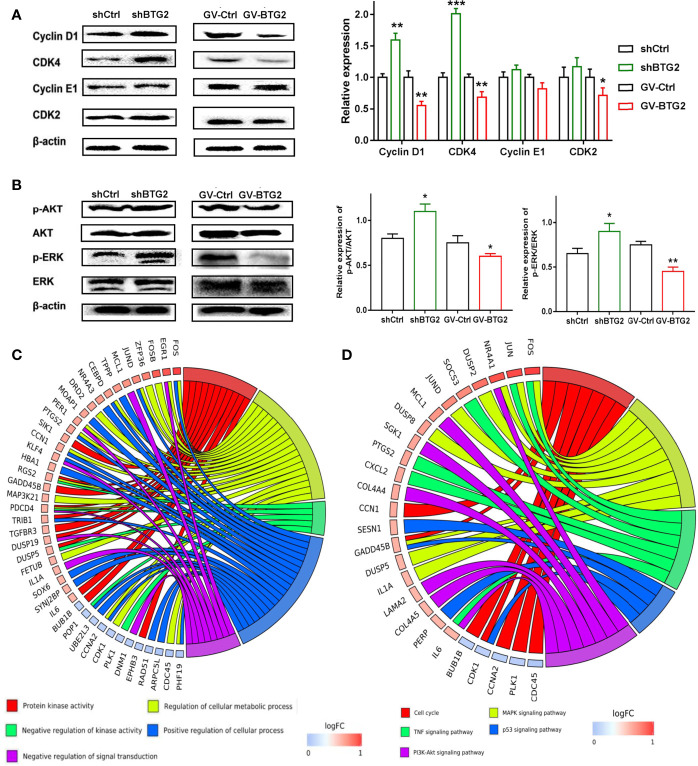
BTG2 inhibited ovarian cancer proliferation through the AKT and ERK-MAPK signaling pathway. **(A)** Protein levels of Cyclin D1, CDK4, Cyclin E1 and CDK2 were determined by Western blot on the left side. The ratios of Cyclin D1, CDK4, Cyclin E1, CDK2, were relative to β-actin and normalized to non-treated control group on the right side. **(B)** Protein levels of p-AKT, AKT, p-ERK1/2 and ERK1/2 were determined by Western blot on the left side and The relative expression of p-AKT/AKT and p-ERK/ERK of shBTG2 cells and GV-BTG2 cells was on the right side. Data were presented as means ± SD of three independent experiments compared to the negative control group. **(C)** GO Chord plot of 5 significantly ranked represented GO terms belonging to the Biological Process subontology for BTG2 downstream genes. **(D)** KEGG Chord plot of 5 significantly ranked represented KEGG pathways for BTG2 relative downstream genes. *p < 0.05, **p < 0.01, ***p < 0.001.

To explore the underlying molecular mechanisms, we further confirmed the expression of genes related to the cell cycle, MAPK signaling and PI3K-AKT signaling activation in the shBTG2 or GV-BTG2 ovarian cancer cells (**Figures 5A, B**). shBTG2 caused a notable increase in the expression of the cell cycle proteins Cyclin D1 and CDK4, while GV-BTG2 decreased Cyclin D1, CDK4 and CDK2 expression in ovarian cancer cells. Our results also showed that phosphorylation levels of AKT and ERK were increased in shBTG2 group and decreased in GV-BTG2 group compared to the respective control groups. Consistent with expression of genes, we performed GO annotation and KEGG pathway enrichment of BTG2 relative downstream genes (**Figures 5C, D** and **Tables S9, S10**). GO annotations included response to protein kinase activity, regulation of cellular metabolic process, negative regulation of kinase activity, positive regulation of cellular process and negative regulation of signal transduction. The top KEGG pathways were response to cell cycle, MAPK signaling pathway, PI3K-AKT signaling pathway, TNF signaling pathway and p53 signaling pathway.

These findings suggested that BTG2 might inhibit ovarian cancer growth by accelerating the cell cycle and activating the MAPK signaling and PI3K-AKT signaling pathways.

### The Effect of BTG2 on Chemosensitivity in Ovarian Cancer Cells

**Figure 6:**
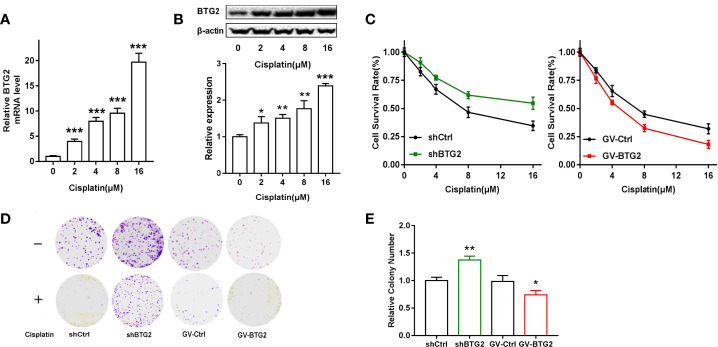
BTG2 impairs the viability and colony formation of cisplatin-treated A2780 cells. **(A)** A2780 cells were treated with cisplatin (0, 2, 4, 8, 16μM) for 48h. Quantitative real-time PCR assay showed mRNA levels of BTG2 after cisplatin treatment. **(B)** A2780 cells were treated with cisplatin (0, 2, 4, 8, 16μM) for 48h Western blot showed protein levels of BTG2 after cisplatin treatment. **(C)** Cisplatin induced cell viability of A2780 cells was detected by MTT assay. **(D)** Cells were treated with or without cisplatin (2μM) for 15 days and the remained colonies were stained and counted. **(E)** The colony number was relative to negative control cells. *p < 0.05, **p < 0.01, ***p < 0.001.

Chemotherapy is the preferred treatment for ovarian cancer, but the 5-year survival rate remains low partly because of the development of drug resistance (29). To investigate the function of BTG2 in drug resistance, we detected the expression of BTG2 following cisplatin treatment. Cisplatin treatment significantly induced BTG2 expression in a dose-dependent manner in A2780 cells, as shown in **Figures 6A, B**. Moreover, MTT and cell colony assay results revealed that cell viability of the shBTG2 cells was significantly higher than that of the shCtrl cells following cisplatin treatment and cell viability of the GV-BTG2-A2780 cells was lower than that of the control cells with the same cisplatin treatment (**Figures 6C–E**). In contrast, the sensitivity to cisplatin treatment of the shBTG2 ovarian cancer cells was weaker than that of the shCtrl group, while the sensitivity of the GV-BTG2 group was stronger than that of the GV-Ctrl group. This result indicated that the inhibition of ovarian cancer cell proliferation induced by cisplatin may be partially regulated by BTG2, and the absence of BTG2 expression may reduce the chemosensitivity of ovarian cancer cells.

### BTG2 Suppresses Tumorigenicity *In vivo* and in the Clinic

**Figure 7:**
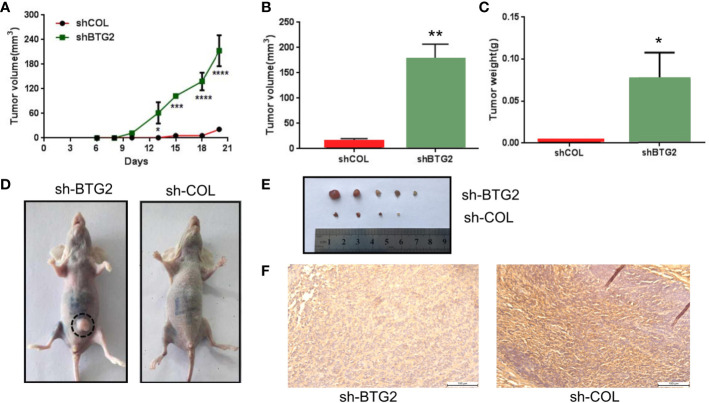
BTG2 suppresses tumorigenicity *in vivo* and in clinic. **(A)** Tumor growth curve of BTG2 knockdown tumors (n = 5) and control tumors (n=5). **(B)** Analysis of tumor volume in BTG2 knockdown group and control group. **(C)** Analysis of tumor weight in BTG2 knockdown group and control group. **(D)** Representative images of tumorigenicity assay performed in nude mice. **(E)** Tumors were obtained from nude mice injected subcutaneously with A2780 cells transfected with sh-BTG2. **(F)** Immunohistochemistry images of mice tumors in X20 microscope in NC-transfected and shBTG2-transfected mice. *p < 0.05, **p < 0.01, ***p < 0.00, ****p < 0.0001.

To investigate the effects of BTG2 expression on tumor growth *in vivo*, we subcutaneously injected BTG2 shRNA and normal control A2780 cells into the posterior ventral side of nude mice. Compared with the NC-transfected A2780 cells, the shBTG2-transfected cells led to an increased tumor volume (p<0.01) (**Figures 7A, B**) and tumor weight (p<0.05) (**Figure 7C**) after tumor formation. As shown in **Figures 7D, E**, BTG2 inhibited NC tumor growth compared to that of the shBTG2 group. Immunohistochemical staining of the xenograft tumors showed lower BTG2 expression in the shBTG2 tumors than in the NC tumors (**Figure 7F**).

## Discussion

Major changes in the expression of tumor suppressor genes are a noteworthy phenomenon in multiple tumor tissues. Here, by comparing tumor samples (ovarian cancer samples from TCGA) to normal samples, we identified a set of 24 downregulated genes that also affected patient survival. We also found most of those 24 tumor suppressors had higher expression in ovarian cancer samples than in lower grade samples, suggesting their potential impact on tumor progression and aggressiveness. To explore mechanisms that could contribute to the downregulation of those genes, we analyzed nonsynonymous mutations and CNAs. Ovarian cancer exhibits a high mutational load compared to other tumor types, and top 5 most frequently mutated genes included BTG2. Furthermore, based on the PPI network, the importance of these 5 genes was evaluated by degree. Overall, BTG2, a member of the BTG/TOB (B-cell translocation gene/transducers of ErbB2) gene family, showed the highest node degree and exhibited the most consistent results in bioinformatics analysis.

To further investigate the effect of BTG2 expression on ovarian cancer progression, we overexpressed and knocked down BTG2 in ovarian cancer cell lines. We found that the proliferation of ovarian cancer cells was inhibited by BTG2 and confirmed the effects and mechanisms of BTG2-sensitized ovarian cancer by establishing nude mouse xenograft models using the BTG2 shRNA- and NC-transfected A2780 cells. The flow cytometry results indicated that BTG2 could induce G1 cell cycle arrest in ovarian cancer cells. Cyclin D1, Cyclin E1, CDK2 and CDK4 are important proteins in the G1 to S stage (30–32). Our results revealed that the expression levels of Cyclin D1 and CDK4 were decreased when BTG2 was overexpressed, but no significant change was found for Cyclin E1 and CDK2. These data implied that BTG2 regulated the cell cycle through Cyclin D1 and CDK in ovarian cancer cells.

We also detected AKT and MAPK pathway activation following aberrant BTG2 expression. AKT can phosphorylate GSK3 to induce the activation of Cyclin D and Cyclin E, which is necessary for the transition from G1 phase to S phase (33, 34). Extracellular regulated protein kinases (ERKs) belong to the mitogen-activated protein kinase (MAPK) family (35)and regulate the cell cycle (36, 37). The results of this study also indicated that BTG2 suppressed the phosphorylation of AKT and ERK, which may result in inhibiting ovarian cancer growth.

Metastasis is a characteristic of malignant tumors and is the main cause of poor prognosis and death in ovarian cancer patients (38). This study showed that BTG2 expression decreased with tumor grade and FIGO stage in ovarian cancer patients. Therefore, we suspected that BTG2 expression is involved in metastasis or migration. Indeed, lower expression of BTG2 increased cell migration in ovarian cancer cells. Metastatic cancer cells can induce the degradation of proteins in the basement membrane and extracellular matrix, which are induced by matrix metalloproteinase (MMP) (39). The results showed that MMP-2 and MMP-9 protein expression in the shBTG2 group was higher than that in the shCOL group. These data suggested that BTG2 inhibited migration by regulating MMP2/9 expression.

Cisplatin is a common compound that has been used in the clinic to treat ovarian cancer (40). More than 70% of the patients with ovarian cancer in the initial cisplatin chemotherapy had a good treatment effect, but cisplatin resistance will occur in most ovarian cancer patients (41–43). It is necessary to improve the effectiveness of chemotherapy. This study found that BTG2 could increase the sensitivity of ovarian cancer cells to cisplatin. The mechanism may be related to BTG2-induced cell cycle arrest (44).

Thus, BTG2 was downregulated in ovarian cancer tissues and associated with patient survival. BTG2 inhibited ovarian cancer cell proliferation and migration, induced cell cycle arrest and enhanced cisplatin sensitivity *in vitro*. Furthermore, we confirmed that BTG2 played a role in tumorigenicity in a xenograft model. This study indicated that BTG2 may be a tumor suppressor and a potential biomarker for ovarian cancer patients.

## Data Availability Statement

The datasets presented in this study can be found in online repositories. The names of the repository/repositories and accession number(s) can be found in the article/**Supplementary Material**.

## Ethics Statement

The studies involving human participants were reviewed and approved by The Ethical Research Committee of the First Affiliated Hospital of Dalian Medical University. The patients/participants provided their written informed consent to participate in this study. The animal study was reviewed and approved by Experimental Animal Ethics Committee of Dalian Medical University (No. AEE19104).

## Author Contributions

WL designed the study. JW, HL, and JiZ contributed to data analysis and visualization. HL, LW, LQ, and JuZ processed the samples. JWand HL performed statistical analyses. JW, LW, ML, JuZ, LL, CZ, and WL contributed to expertise and invaluable critical discussion. JW and HL wrote the article. JG procured the pathology slides. All authors contributed to the article and approved the submitted version.

## Funding

This study was supported by the National Natural Science Foundation of China (Grant No. 81302282 to WL), Natural Science Foundation of Liaoning Province (Grant No. 20170540273 to WL) and the Education fund item of Liaoning Province (Grant No. 2017002 to WL). China Postdoctoral Science Foundation (Grant No.2020M680920 to JW).

## Conflict of Interest

The authors declare that the research was conducted in the absence of any commercial or financial relationships that could be construed as a potential conflict of interest.

## Publisher’s Note

All claims expressed in this article are solely those of the authors and do not necessarily represent those of their affiliated organizations, or those of the publisher, the editors and the reviewers. Any product that may be evaluated in this article, or claim that may be made by its manufacturer, is not guaranteed or endorsed by the publisher.
